# Exploring segmented assimilation theory in health education utilization and its influencing factors among internal migrants in China: insights from the 2017 China migrants dynamic survey

**DOI:** 10.3389/fpubh.2024.1529736

**Published:** 2025-01-08

**Authors:** Ting Xu, Zeyu Wang, Tingting Wang, Jiahua Shi, Aiyong Zhu, Enhong Dong

**Affiliations:** ^1^School of Nursing and Health Management, Shanghai University of Medicine and Health Sciences, Shanghai, China; ^2^Institute of Healthy Yangtze River Delta, Shanghai Jiao Tong University, Shanghai, China; ^3^College of Medical, Veterinary & Life Sciences, University of Glasgow, Glasgow, United Kingdom; ^4^Huangpu District Health Promotion Center, Shanghai, China

**Keywords:** health education utilization, segmented assimilation, internal migrants, acculturation, China

## Abstract

**Introduction:**

This study investigated segmented assimilation patterns and factors influencing health education utilization (HEU) among internal migrant populations in China, driven by concerns over their declining health owing to urbanization-related changes.

**Methods:**

Data from the 2017 China Migrants Dynamic Survey were analyzed, focusing on 13,998 rural migrants. Negative binomial regression was used to explore assimilation patterns and determine the factors affecting HEU among internal migrants in China.

**Results:**

The results revealed diverse assimilation patterns among internal migrants in four clusters: first-generation classic assimilation, first-generation integration assimilation, second-generation segmented assimilation, and second-generation underclass assimilation. Adjusting for socioeconomic factors, first-generation integrated assimilation groups showed lower HEU (IRR = 0.922, *p* < 0.01), while second-generation underclass groups demonstrated higher HEU (IRR = 1.110, *p* < 0.001) than the second-generation segmented assimilation groups. Additionally, factors such as ethnicity, marital status, employment status, educational attainment, hukou type, health insurance type, time of access to healthcare, social integration, social participation, establishment of health records, and issues encountered in host and origin places significantly influenced HEU.

**Discussion:**

This study highlights diverse assimilation patterns among Chinese internal migrants regarding HEU, consistent with the theory of segmented assimilation. Specifically, second-generation immigrants exhibit higher HEU levels than their first-generation counterparts, with the second-generation underclass demonstrating the highest HEU. These findings underscore the need for targeted policy interventions addressing diverse migrant assimilation patterns. Specifically, first-generation migrants require accessible and culturally adapted health education programs to overcome systemic barriers, while second-generation underclass migrants need sustained support to leverage their engagement in health initiatives.

## 1 Introduction

Most developed countries, particularly the United States, recognize the critical role of health education in reshaping individual health behaviors, preventing diseases, improving overall population health, and managing healthcare expenditures (1). Growing immigrant populations in regions such as North America and Europe have intensified academic attention on immigrants’ health conditions and adaptability, highlighting the importance of research in this area (2, 3). Research indicates that many immigrants arrive with insufficient health education and basic health literacy, contributing to a heightened risk of chronic and infectious diseases and, consequently, a gradual decline in overall health (4). This trend is particularly evident in birth outcomes, as first-generation immigrant women give birth to healthy children whose subsequent generations show comparatively poorer health outcomes (5). Various studies have substantiated the effectiveness of health education in improving immigrant health. For instance, Ponce-Gonzalez et al. (6) demonstrated that community workers successfully increased flu vaccination rates among low-income Latin immigrants through continuous and diverse health education initiatives. Similarly, in Washington State, community workers provided sustained oral health education to immigrants and refugees, leading to lasting improvements in oral health knowledge and practices within these communities (7).

Previous research has identified several challenges in designing and implementing health education initiatives for immigrants, including socioeconomic status, educational background, legal immigration status, cultural background, language proficiency, health literacy, acculturation, and lifestyle (1, 8). For high-income or documented immigrants, factors such as cultural adaptation, adjustments to the new healthcare system, differences in health beliefs, and time constraints significantly affect health education programs. In contrast, low-income or undocumented immigrants encounter barriers like language obstacles, economic conditions, and lower educational attainment, which hinder their access to health education services (9). Many immigrants live in poverty, resulting in second-generation children rarely having access to government-supported children’s health programs, including health education and nutritional support initiatives (10). This situation poses significant challenges to the intergenerational health of immigrants in countries like the United States (11).

Contemporary research on immigrant health education primarily addresses obstacles, strategies, and factors influencing overall health education for immigrants. However, a notable gap exists in studies examining the integration of health education with the acculturation processes experienced across immigrant generations (1, 12, 13).

Segmented assimilation (SA) theory, first proposed by Portes et al. (14), outlines four distinct pathways of social and economic adaptation among immigrants, each associated with unique health outcomes. These include: (1) upward assimilation, where integration into the host society’s middle or upper classes and adoption of dominant cultural values often enhance health outcomes through improved socioeconomic conditions; (2) downward assimilation, marked by socioeconomic decline and integration challenges, which are frequently associated with poorer health due to stress and limited resources; (3) selective acculturation, where immigrants maintain aspects of their ethnic identity while partially integrating into the host society, resulting in mixed health outcomes influenced by both traditional and new practices; and (4) integrated assimilation, which blends classic assimilation with multiculturalism, promoting positive health behaviors by leveraging cultural adaptability and economic advantages. Previous study provide evidence for these trajectories, showing correlations in socioeconomic status and improved health outcomes, such as greater life satisfaction (15).

Building on SA theory, immigrants often experience initial health advantages over local populations, referred to as segmentation, before gradually assimilating into the host population’s health norms (16). This process involves lifestyle adjustments, belief shifts, and behavioral changes influenced by cultural adaptation. Intergenerational dynamics further complicate assimilation, with second-generation immigrants navigating conflicting values between host and origin cultures, often leading to heightened identity struggles, discrimination, and negative health outcomes (17–19). Examining the application of SA to health education among migrants is vital given its impact on health outcomes, underscoring the necessity for targeted strategies to address their diverse needs.

During China’s reform and opening-up, rapid economic growth and urbanization led to a significant increase in internal migration, with approximately 376 million migrants by 2020, accounting for 27% of the population (20, 21). Despite living in areas with better medical resources, internal migrants often experience substantially lower healthcare utilization than local residents (11, 22), driven by barriers such as limited health literacy, inadequate social integration, and disparities in healthcare accessibility. These issues contribute to widening health inequities, exacerbating the health risks faced by migrant populations. Drawing parallels with international migration, this study used data from the 2017 China Migrants Dynamic Survey (CMDS) and applied a negative binomial regression model to examine how health education can improve healthcare utilization among internal migrants. Additionally, this study investigates the segmentation assimilation dynamics of health education among immigrants, offering insights for future research and policy interventions.

### 1.1 Research hypotheses

**Figure 1:**
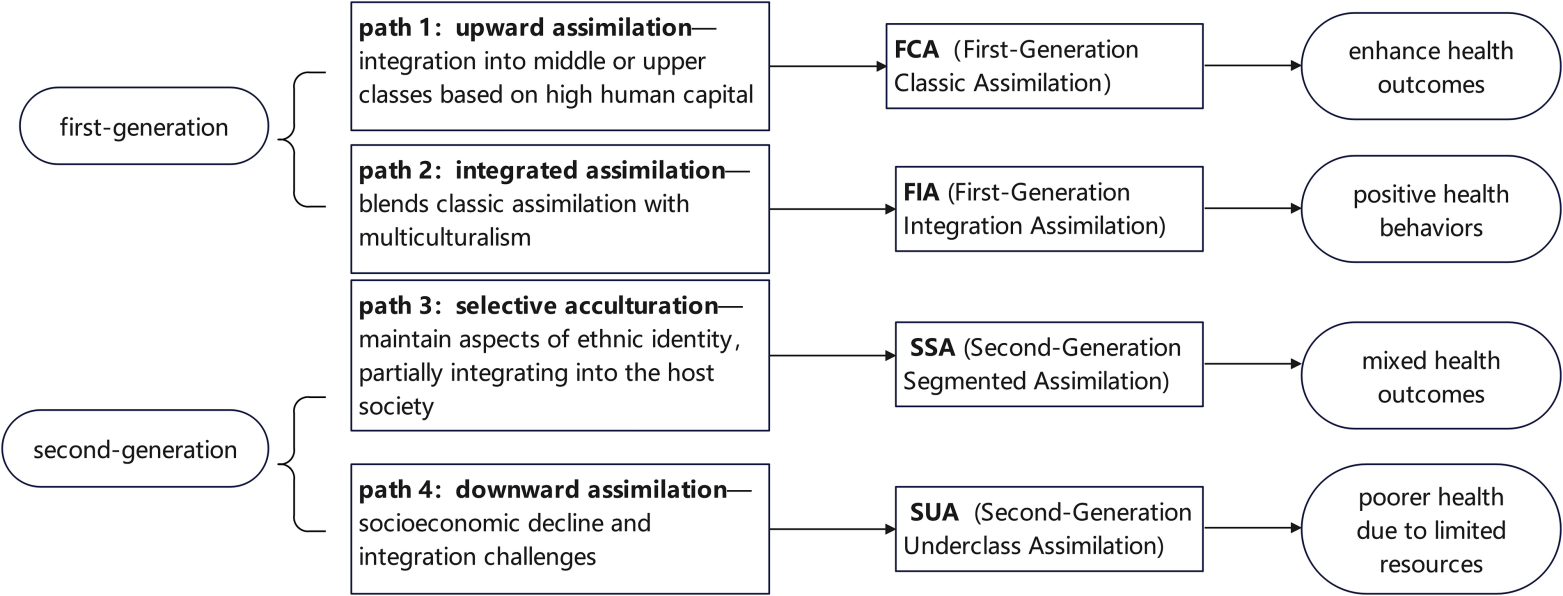
Conceptual framework of segmented assimilation trajectories and health outcomes in internal migrants.

Several studies have validated Portes’ et al. (14) four SA pathways for first- and second-generation migrants. For instance, Lee et al. (23) surveyed 356 Korean Americans using Gordon’s theoretical framework and identified three immigrant types: acculturated, bicultural, and traditional, confirming the multidimensional nature of the acculturation-health relationship. Flannery et al. (24) analyzed 291 Asian Americans and identified four cultural adaptation types: traditional (separation), assimilation, integration (biculturalism), and marginalization. Ramírez et al. (16) categorized Latino immigrants in NHANES data (2007–2016) into three groups: classic, underclass, and selective, highlighting the SA of dietary health. Karimi and Wilkes (25) systematically reviewed recent studies on classic, segmented, and neo-assimilation theories that define immigrants’ assimilation trajectories. Building on SA theory and these findings, this study identified four assimilation types: First-Generation Classic Assimilation (FCA), First-Generation Integration Assimilation (FIA), Second-Generation Segmented Assimilation (SSA), and Second-Generation Underclass Assimilation (SUA) (see Figure 1). We propose that SA is also present among immigrant health education utilization (HEU) in China, leading to Hypothesis 1:

Hypothesis 1: Internal Chinese migrants exhibit various patterns of assimilation in HEU, supporting the SA theory.

Previous studies employing SA theory have shed light on the relationship between acculturation and immigrant engagement in health education (26–28). First-generation immigrants often face challenges adapting to the North American healthcare consumption model, which emphasizes individual health responsibility (29). Regarding Chinese internal migration, the inflow of rural migrants into major cities with abundant medical resources presents significant challenges, particularly as they may be unfamiliar with concepts such as proactive health management. In this context, FCA immigrants, who possess higher education levels and economic stability, tend to integrate into the host country’s middle- and upper-class societies. They actively adapt to the North American healthcare system, demonstrating a strong inclination to minimize excessive healthcare expenditures and time investments, indicating a high acceptance of health education. Consequently, FCA immigrants exhibit a robust commitment to navigating the North American healthcare system effectively, further supporting their acceptance of health education (25). Therefore, we propose Hypothesis 2a.

Hypothesis 2a: The FCA type has a higher acceptance of HEU than the SSA type.

The FIA group generally exhibits good physical health but relatively lower educational attainment and socioeconomic status. A primary motivation for migration in this group is to increase income and enhance living standards. They typically devote significant time and energy to work for financial gain (30) and usually adopt an integrative approach to the cultures of both their host and original countries. Health education and access to medical services are not primary concerns for this group (9). Furthermore, their lower educational attainment presents challenges in obtaining information, leading them to seek medical assistance from their place of origin when navigating an unfamiliar healthcare system (29). Consequently, their propensity to engage in health education is relatively low (1), prompting us to formulate Hypothesis 2b.

Hypothesis 2b: The FIA type has less acceptance of HEU than the SSA type.

Empirical evidence indicates that socioeconomic disparities significantly influence the reception of health education among second-generation immigrants, irrespective of their familiarity with the host country’s medical infrastructure or language proficiency. Specifically, socially and economically disadvantaged SUA immigrants are more likely to engage in health education programs. This inclination stems from financial limitations and a heightened awareness of the barriers to healthcare accessibility (6.7.32). Based on the SA theory, which explains how immigrant communities adopt new values and change behaviors, we propose Hypothesis 2c.

Hypothesis 2c: The SUA type has a higher acceptance of HEU than the SSA type.

## 2 Materials and methods

### 2.1 Data sources

This study used data from the 2017 CMDS database administered by the National Health Commission of China (NHFPC). This database encompasses a nationally representative cross-sectional survey spanning 31 provinces, municipalities, autonomous regions, and areas with floating populations.

Inclusion criteria were as follows: (1) individuals aged 15–59 years, a widely adopted standard that facilitates comparison with previous studies; (2) members of the domestic floating population who had resided in an immigrant city for over 1 month, consistent with the criteria established by the NHFPC when it initiated the survey in 2009 to track migrants’ living conditions; and (3) exclusion of cases with missing data on key variables, such as education, income, and medical insurance.

The survey methodology involved two main approaches: (1) a proportional sampling method (PPS), which facilitated the selection of eight provinces (including municipalities and autonomous regions)—Jiangsu, Guangdong, Shandong, Henan, Hunan, Yunnan, Xinjiang, and Chongqing—based on the demographic characteristics of domestic immigrants in each province. This method ensured a high representativeness of the data and a comprehensive range of variables. (2) A stratified multistage sampling method was employed to target the floating population who had resided in their destination for over 1 month and lacked local household registration. Trained interviewers, supervised by health committee staff in each survey area, conducted face-to-face interviews.

Following a preliminary analysis of the database sample, the study included 13,998 internal migrants from the survey to examine the patterns of HEU among Chinese immigrants in their destination regions.

The secondary data utilized in this research was obtained from the CMDS, which is officially released by the NHFPC and includes information from 31 provinces (as well as municipalities and autonomous regions) spanning the years 2009 to 2017. The original data that supports the results of this study can be requested from the corresponding author with a valid request. Since this study relied on secondary data, there was no requirement for participation from patients or the public, particularly regarding ethical issues.

### 2.2 Measurement

#### 2.2.1 HEU

The central explanatory variable in this study is HEU. This variable was assessed based on responses to 15 questions covering two main dimensions: the modality and content of health education received by participants in the past year. The health education content comprised nine questions on occupational disease prevention, STD/AIDS prevention and control, reproductive health and contraception, tuberculosis prevention and control, smoking control, mental health, chronic disease prevention and control, maternal and child health, and emergency self-rescue.

Health education methods were assessed through six questions measuring the participants’ exposure to various health education channels, including health knowledge lectures, promotional materials, public health information displays, community health consultation events, SMS/WeChat/website platforms, and personalized face-to-face consultations. Participants provided binary responses, with “YES” coded as 1 and “NO” coded as 0.

#### 2.2.2 Acculturation

This study developed a cultural fitness measurement method using a two-dimensional model to assess variables. This measurement focuses on the degree of acculturation, which is evaluated using two dimensions: host culture integration and origin culture maintenance.

Host culture integration comprised four items, such as “I desire to assimilate and integrate into the local community.” Respondents rated each item on a scale ranging from “strongly disagree = 1” to “strongly agree = 4,” with higher scores indicating a greater capacity to adapt to the host culture.

Original culture maintenance encompassed three items, including “My hygiene practices significantly differ from those of local residents.” Participants rated each item using the same scale, from “strongly disagree = 1” to “strongly agree = 4,” where higher scores signify a greater commitment to preserving the original cultural norms and practices.

#### 2.2.3 Period of residence (POR)

Period of residence was categorized into four intervals: “1–5 years” (coded as 1); “6–10 years” (coded as 2); “11–20 years” (coded as 3); and “≥20 years” (coded as 4).

#### 2.2.4 SA patterns

Before conducting the k-means cluster analysis, all variables were standardized to address potential discrepancies in standard deviations and means. Continuous variables were transformed into *z*-scores (mean = 0, standard deviation = 1) to ensure consistency across measurement scales. Variable selection was guided by an SA framework, emphasizing socioeconomic position, social acculturation, and generation status.

Socioeconomic position variables included monthly income (continuous) and education level (categorical), while acculturation-related variables comprised host culture integration (sum of five items) and origin culture maintenance (sum of three items). Following the literature (31, 32), international immigrants were categorized into first- and second-generation groups, with the latter defined as individuals with at least one parent born abroad. Generation status was further classified into first-generation migrants (born before 1980) and second-generation migrants (born in or after 1980).

These variables were utilized in a two-step clustering procedure to identify distinct assimilation patterns, which were subsequently classified into four categories: FCA, FIA, SSA, and SUA.

#### 2.2.5 Social integration and social participation

Social integration was measured using six items asking if respondents had participated in the activities of local organizations (e.g., labor unions and volunteer associations) since 2016, rated 0 (No) or 1 (Yes). Higher scores indicated greater integration. Social participation was measured with five items assessing involvement in village or community management (e.g., reporting situations to government departments), rated from 0 (none) to 4 (often), with higher scores indicating greater participation. In this study, Cronbach’s *α* was 0.876 for social integration and 0.896 for social participation.

#### 2.2.6 Covariates

The covariates considered in this study included demographic variables (age, sex, ethnicity, marital status, and employment status), socioeconomic variables (educational attainment, monthly income, household registration status, and health insurance type), time taken to access medical services, establishment of health records, challenges encountered in the host country, challenges encountered in the original country, social integration, and social participation. Table 1 provides concise definitions and the corresponding values assigned to these covariates for ease of reference.

**Table 1:** Assignment definitions and values.

1. Continuous variable	Measurement	2. Categorical variable	Measurement and assignment
HEU	/	Segmented assimilation type	First Classical Assimilation type = 1; Integrated assimilation type = 2;Segmented Assimilation type = 3; Underclass Assimilation type = 4
Age	/	Acculturation	Host culture integration^a^: from “strongly disagree = 1” to “strongly agree = 4”. Origin culture maintenance^b^: from “strongly disagree = 1” to “strongly agree = 4”
Monthly Income	/	Gender	Male = 1; Female = 2
Problems encountering in host place	/	Ethnicity	Han^c^ = 1; Minority = 0
Problems encountering in original place	/	Marital status	Married = 1; Unmarried = 0
Social integration	/	Employment status	Employed with a fixed employer = 1; Employed with unfixed employer = 2; employer = 3; self-employed worker = 4; others = 5
Social participation	/	Insurance type (NRCMS,URRBMI,URBMI,UEBMI,FI)*	Yes = 1; No = 0
		Educational attainment	Illiteracy = 1; primary school = 2; junior high school = 3;high school/technical secondary school^d^ = 4; junior college^e^ = 5; undergraduate college = 6; graduate = 7
		Household registration type (Hukou Type)**	Agricultural = 1; non-agricultural = 2; agricultural to residential = 3; Non-agricultural to residential = 4; residential = 5; others = 6
		Time of Access to health services	Within 15 min = 1; 15–30 min = 2; 30–60 min = 3; more than 60 min = 4
		Establishment of health record	Yes = 1; No = 0
		POR	1–5 years = 1; 5–10 years = 2; 10–20 years = 3; more than 20 years = 4

*NRCMS, New Rural Cooperative Medical Scheme; URRBMI, Urban and Rural Residents Basic Medical Insurance; URBMI, Urban Resident Basic Medical Insurance; UEBMI, Urban Employee Basic Medical Insurance; FI, free medical insurance.

**The Hukou system is a distinctive population management system implemented in China to efficiently oversee the domestic population and regulate population movement.

^aHost culture integration reflects a desire to assimilate and actively engage with the local community.^

^bOrigin culture maintenance signifies preserving cultural practices distinct from those of the local community.^

^cThe Han ethnic group, over 92% of China’s population, is the largest in the country.^

^dTechnical secondary school provides vocational training for students aged 15–18, focusing on specific skills in fields like engineering, health care, and business in China.^

^eJunior college offers 2–3 years of post-secondary education, emphasizing practical and technical skills. Graduates receive a diploma and can either enter the workforce or transfer to a university for further study.^

### 2.3 Analytical strategy

K-means cluster analysis was applied to examine whether internal migrants could be classified into four SA patterns based on educational attainment, annual income, and acculturation (host culture integration and origin culture maintenance) as essential characteristics of the distinct assimilation patterns in the cluster analysis. This method was chosen for its simplicity, flexibility, computational efficiency, and ease of implementation. Its proven effectiveness with large datasets and widespread application across various domains further underscores its suitability, making it a more practical choice than alternatives such as hierarchical clustering (33).

Furthermore, a one-way analysis of variance (ANOVA) was performed for multiple variables, including SA type, employment status, educational attainment, hukou type (urban or rural), and marital status. An independent samples t-test was conducted for the binary independent variables [e.g., educational attainment, hukou type, participation in the New Rural Cooperative Medical System (NRCMS), establishment of health records, and challenges encountered in the host and origin countries]. Covariates that were statistically significant in the one-way ANOVA or *t*-test were retained for subsequent multivariate regression analyses.

Given that the dependent variable, HEU, is a count outcome variable, Poisson regression or negative binomial regression could be employed to examine the effects of confounding and predictor variables. Negative binomial regression models count data, especially when events are rare or uneven, and is more flexible than Poisson regression, which assumes equal mean and variance. It is used when the variance exceeds the mean, handling overdispersed data better. The analysis revealed that the mean count (3.645) was significantly lower than the variance (10.840), indicating that the variance substantially exceeded the mean. Furthermore, a likelihood ratio test for the alpha parameter yielded a chi-squared value of 3844.12 with one degree of freedom. This result strongly indicates that alpha is non-zero, suggesting that the negative binomial model is more suitable than the Poisson model for this analysis (see Appendix 1 for details on the Poisson regression results). Since the *p*-value of the test was less than the significance level (e.g., 0.05), we concluded that the negative binomial regression model offers a significantly better fit.

### 2.4 Statistical software

All analyses were conducted using Stata 15.1 (Stata Corp LP, College Station, TX, United States). All tests were two-tailed, with significance level set at *p* < 0.05 for *t*-tests, one-way ANOVA, and negative binomial regression analyses.

## 3 Results

### 3.1 Descriptive analysis

Tables 2, 3 present the descriptive statistics for the 13,998 valid responses. The average age of respondents is 35.20 years (±10.25), with a mean monthly income of $1,025.83 (±715.13). Most participants were male (7,163, 51.17%), of Han ethnicity (12,632, 90.24%), married (11,458, 81.85%), and had completed junior middle school education (5,815, 41.54%). The sample’s demographic characteristics, including age, sex, and marital status, were consistent with findings from previous studies (34), supporting the representativeness and suitability for analysis.

**Table 2:** General Characteristics of the participants and one-way ANOVA analysis (*n* = 13,998).

Values	Frequency (%)	HEU	*F*-*/p-*value
		(M ± SD)	
Segmentation assimilation type			*F* = 2.93, *p* = 0.022
FCA	630 (4.5)	3.46 ± 3.21	
FIA	4,575 (32.68)	3.27 ± 3.19	
SAA	5,396 (38.55)	3.42 ± 3.32	
SUA	3,397 (24.27)	3.38 ± 3.32	
POR			*F* = 52.49, *P* < 0.001
1–5 years	9,221 (65.87%)	3.11 ± 3.24	
5–10 years	2,685 (19.18%)	3.90 ± 3.26	
10–20 years	1754 (12.53%)	3.74 ± 3.40	
More than 20 years	338 (2.41%)	3.87 ± 3.61	
Gender			*t* = 0.23, *p* = 0.8124
Male	7,163 (51.17%)	3.37 ± 0.04	
Female	6,835 (48.83%)	3.36 ± 0.04	
Ethnicity			*t* = 5.25, *P* < 0.001
Han	12,632 (90.24%)	3.31 ± 0.03	
Minority	1,366 (9.76%)	3.84 ± 0.10	
Marital status			*t* = 7.64, *P* < 0.001
Married	11,458 (81.85%)	3.46 ± 0.03	
Unmarried	2,540 (18.15%)	2.91 ± 0.07	
Employment status			*F* = 7.50, *P* < 0.001
Employee with a fixed employer	6,225 (44.50%)	3.24 ± 3.32	
Employee with unfixed employer	761 (5.40%)	3.24 ± 3.48	
Employer	641 (4.62%)	3.52 ± 3.29	
Self-employed worker	4,185 (29.9%)	3.56 ± 3.19	
Others	177 (1.31%)	3.88 ± 3.66	
Educational attainment			*F* = 12.55, *P* < 0.001
Illiteracy	269 (1.92%)	2.17 ± 2.94	
Primary school	1,604 (11.46%)	3.10 ± 3.32	
Junior high school	5,815 (41.54%)	3.30 ± 3.23	
High school/technical secondary school	3,710 (26.50%)	3.44 ± 3.27	
Junior college	1,690 (12.07%)	3.64 ± 3.41	
Undergraduate	865 (6.18%)	3.76 ± 3.41	
Graduate	45 (0.32%)	3.58 ± 3.48	
Hukou type			*F* = 8.65, *P* < 0.001
Rural household registrations	11,619 (83.00%)	3.30 ± 3.29	
Non-rural	1,557 (11.12%)	3.77 ± 3.41	
Rural to residential	445 (3.18%)	3.86 ± 2.98	
Non-rural to residential	42 (0.30%)	3.81 ± 2.62	
Residential	330 (2.36%)	2.97 ± 3.22	
Others	5 (0.04%)	3.40 ± 4.28	
Insurance type			
NRCMS			*t* = 5.67, *P* < 0.001
Yes	8,650 (61.80%)	3.24 ± 0.03	
No	5,348 (38.20%)	3.57 ± 0.05	
URRBMI			*t* = −6.45, *P* < 0.001
Yes	814 (5.82)	4.13 ± 0.12	
No	13,184 (94.18)	3.32 ± 0.29	
URBMI			*t* = −5.02, *P* < 0.001
Yes	770 (5.50%)	3.94 ± 0.12	
No	13,228(%)	3.33 ± 0.29	
UEBMI			*t* = −2.90, *P* = 0.0038
Yes	3,315 (23.7%)	3.51 ± 0.06	
No	10,683 (76.3%)	3.32 ± 0.03	
FI			*t* = −9.94, *P* < 0.001
Yes	211 (1.5%)	5.60 ± 0.24	
No	13,787 (98.5%)	3.33 ± 0.28	
Time of access to health care			*F* = 7.42, *P* < 0.001
Within 15 min	11,729 (83.8%)	3.35 ± 3.29	
15–30 min	1956 (14%)	3.39 ± 3.27	
30–60 min	275 (2%)	3.62 ± 3.51	
More than an hour	38 (0.3%)		
Establishment of health record			*t* = −34.31, *p* < 0.001
Yes	3,925 (31.46%)	5.19 ± 0.05	
No	8,551 (68.54%)	3.13 ± 0.03	

**Table 3:** Correlation analysis with health education utilization (*n* = 13,998).

Variables	M ± SD	Coefficient of correlation with HEU (*r*)	*P-*value
Age	35.20 ± 10.25	0.018	<0.05
Monthly income	$1025.83 ± 715.13	−0.003	0.700
Problems encountering in host place	8.59 ± 7.17	−0.031	<0.001
Problems encountering in original place	6.08 ± 6.39	0.002	<0.001
Social integration	0.79 ± 1.03	0.235	<0.001
Social participation	5.92 ± 1.40	0.217	<0.001

Most respondents possess rural household registrations (11,619, 83.00%), were covered by the NRCMS (8,650, 61.80%), and had stable employment (6,225, 44.50%). Regarding access to healthcare services, 11,729 respondents (83.8%) reported being able to access services within a 15-min walk. However, 8,511 respondents (68.54%) lacked personal health records. Further details are provided in Tables 2, 3.

Table 4 presents the distribution of the four SA types identified in section 2.2.4. Specifically, the FCA, FIA, SSA, and SUA types comprised 630 (4.50%), 4,575 (32.68%), 5,396 (38.55%), and 3,397 (24.27%) respondents, respectively.

**Table 4:** Mean and Standard deviation (in parentheses) of four patterns of segmented assimilation (*n* = 13,998).

Variables	Cluster			
	FCA	FIA	SSA	SUA
Education attainment	4.03 (1.21)	3.77 (1.11)	3.44 (1.02)	3.35 (1.16)
Monthly Income	24980.95 (8394.09)	10042.24 (2171.68)	5834.56 (859.42)	3127.18 (888.01)
The adaption of one’s host culture	16.68 (2.36)	16.26 (2.41)	16.09 (2.42)	16.05 (2.51)
The maintenance of one’s original culture	6.31 (1.59)	6.47 (1.56)	6.57 (1.59)	6.56 (1.63)

### 3.2 Results of univariate analysis

The *t*-test and one-way ANOVA results revealed significant associations between several categorical variables and HEU. These variables include SA type (*F* = 2.93, *p* = 0.022), POR (*F* = 52.49, *p* < 0.001), ethnicity (*t* = 5.25, *p* < 0.001), marital status (*t* = 7.64, *p* < 0.001), employment status (*F* = 7.50, *p* < 0.001), educational attainment (*F* = 12.55, *p* < 0.001), hukou Type (*F* = 8.65, *p* = 0.001), and insurance types—including NRCMS (*t* = 5.67, *p* < 0.001), urban–rural resident basic medical insurance (URRBMI) (*t* = 6.45, *p* < 0.001), URBMI (*t* = −5.02, *p* < 0.001), UEBMI (*t* = −2.90, *p* = 0.0038), and FI (*t* = −9.94, *p* < 0.001). Additionally, significant associations were found for residential status (*F* = 22.65, *p* < 0.001), time to access healthcare (*F* = 7.42, *p* < 0.001), and the establishment of health records (*t* = −34.31, *p* < 0.001). Further details are provided in Table 2.

Correlation analysis of continuous variables revealed significant associations with HEU. These variables include age (*r* = 0.018, *p* < 0.05), problems encountered in the host place (*r* = −0.031, *p* < 0.001), problems encountered in the original place (*r* = 0.002, *p* < 0.001), social integration (*r* = 0.235, *p* < 0.001), and social participation (*r* = 0.217, *p* < 0.001). Detailed results are provided in Table 3.

### 3.3 Results of negative binomial regression

After adjusting for all significant socioeconomic and other covariates, the incidence rate ratio (IRR) of HEU for individuals categorized under the FIA type was 0.922 (*p* = 0.003), while that for the SUA type was 1.110 (*p* < 0.001). This result suggests that individuals classified under the FIA type were 0.922 times less likely to engage in health education activities than those under the SSA type. In contrast, those classified under the SUA type were 1.110 times more likely.

Additionally, variables such as Han nationality (IRR = 0.887, *p* < 0.01), unmarried status (IRR = 0.871, *p* = 0.001), self-employed status (IRR = 1.118, *p* = 0.001), educational attainment (from primary school to undergraduate level) (IRR > 1.000, *p* < 0.01), hukou type (rural to residential) (IRR = 1.158, *p* < 0.01), URRBMI (IRR = 1.067, *p* = 0.003), URBMI (IRR = 1.130, *p* = 0.003), time required to access healthcare (over an hour) (IRR = 1.534, *p* < 0.001), social integration (IRR = 1.143, *p* < 0.001), social participation (IRR = 1.086, *p* < 0.001), establishment of health records (IRR = 1.196, *p* < 0.001), and issues encountered in host (IRR = 0.581, *p* < 0.001) and origin places (IRR = 0.593, *p* < 0.001) were all significantly associated with favorable HEU. In contrast, variables such as POR, specific aspects of employment status (e.g., employees without fixed employment, employers, and others), educational attainment (e.g., graduate-level education), certain hukou types (e.g., non-agricultural, non-agricultural to residential, residential), FI, and time required to access healthcare (e.g., less than an hour) were not significantly associated with favorable HEU outcomes (*p* > 0.05). Detailed results are presented in Table 5.

**Table 5:** Negative binomial regression analysis of health education utilization among internal migrants in China.

Variables	HEU-IRR value	95% Confidence Interval	*P*-value
Segmentation assimilation type			
SSA type	1.000		
FCA type	0.952	(0.853–1.061)	0.374
FIA type	0.922**	(0.874–0.972)	0.003
SUA type	1.110***	(1.0439–1.185)	<0.001
POR			
1–5 years	1.000		
5–10 years	1.043	(0.988,1.103)	0.126
10–20 years	0.989	(0.918–1.065)	0.768
More than 20 years	1.110	(0.951–1.296)	0.184
Age	0.999	(0.996–1.002)	0.808
Han nationality			
No	1.000		
Yes	0.887***	(0.814–0.966)	<0.001
Marital status			
Married	1.000		
Unmarried	0.871***	(0.806–0.942)	0.001
Employment status			
An employee with a fixed employer	1.000		
An employee without a fixed employer	1.097	(0976–1.232)	0.119
Employer	0.999	(0.899–1.110)	0.985
Self-employed worker	1.118***	(1.053–1.186)	<0.001
Others	1.084	(0.886–1.328)	0.432
Educational attainment			
Illiteracy	1.000		
Primary school	1.385**	(1.047–1.833)	0.002
Junior high school	1.553**	(1.18–2.048)	0.002
High school/technical secondary school	1.660***	(1.255–2.197)	<0.001
Junior college	1.693***	(1.272–2.254)	<0.001
Undergraduate	1.678***	(1.249–2.252)	0.001
Graduate	1.059	(0.640–1.754)	0.821
Hukou type			
Rural	1.000		
Non-agricultural	1.021	(0.943–1.106)	0.607
Agricultural to residential	1.158**	(1.054–1.273)	0.002
Non-agricultural to residential	0.939	(0.751–1.176)	0.588
Residential	0.941	(0.817–1.085)	0.405
Health insurance type			
URRBMI			
No	1.000		
Yes	1.067**	(0.959–1.187)	0.003
URBMI			
No	1.000		
Yes	1.130**	(1.010–1.265)	0.003
UEBMI			
No	1.0000		
Yes	0.882**	(0.815–0.954)	0.002
FI			
No	1.000		
Yes	1.182	(0.975–1.432)	0.088
Time of Access to health care			
Within 15 min	1.000		
15–30 min	1.053	(0.987–1.123)	0.120
30–60 min	0.998	(0.857–1.163)	0.982
More than an hour	1.534***	(1.236–1.904)	<0.001
Social integration	1.143***	(1.112–1.166)	<0.001
Social participation	1.086***	(1.070–10,103)	<0.001
Whether to establish a health record			
No	1.000		
Yes	1.196***	(1.040–1.374)	<0.001
Whether to encounter problems in local place			
No	1.000		
Yes	0.581***	(0.503–0.672)	<0.001
Whether to encounter problems in origin place			
No	1.000		
Yes	0.593***	(0.518–0.678)	<0.001
Constant	45.23	(25.80–79.29)	
Observed value	10,662		
Pseudo *R*-squared	0.1118		
Fitness of model	Pilsenka square value	11196.91	
	*P*-value	0.6689	

*p < 0.05, **p < 0.01, ***p < 0.001.

IRR, Incidence Rate Ratio.

## 4 Discussion

First, the validation of Hypothesis 1 demonstrates that rural Chinese migrants exhibit various forms of SA in HEU. This finding supports the core principle of the SA theory, which posits that immigrants navigate diverse SA pathways influenced by multiple factors during their integration process. Four distinct types of SA (FCA, FIA, SSA, and SUA) were identified. This conclusion is consistent with some international studies (15–18). For instance, Castro et al. (15) categorized immigrants into four distinct trajectory groups based on variations in their socioeconomic status and levels of assimilation, providing empirical evidence to support the SA theory.

Second, the validation of Hypothesis 2b reveals that the FIA group engages less in HEU than the SSA group. According to SA theory, while the FIA group demonstrates a positive integrative attitude toward both their host and original cultures, their primary focus as first-generation immigrants typically revolves around income generation and economic stability. This emphasis often limits their ability to prioritize health education, especially within the host country’s healthcare system, where they encounter several cultural and systemic barriers. These include time constraints due to demanding work schedules, cultural differences in health beliefs, and a lack of familiarity with available health education resources. Information asymmetry further exacerbates these challenges, as FIA immigrants may not have access to or knowledge of health education programs tailored to their needs (35). Many FIA immigrants originate from underdeveloped regions with limited exposure to health education and arrive in urban areas that emphasize proactive health management. This disparity creates a disconnect, as they often lack habits or awareness related to utilizing health education services. Language barriers and cultural stigma around seeking healthcare further hinder their engagement, making them more inclined to seek medical assistance from their place of origin, where they perceive healthcare as more accessible and culturally aligned with their expectations (36, 37). Addressing these systemic and cultural barriers is crucial to increasing HEU participation among FIA immigrants and ensuring equitable access to health resources.

Additionally, first-generation immigrants generally arrive in their host country in good health (38, 39), contributing to their lower HEU rates than SSA immigrants. This finding is consistent with existing literature (40–42). For instance, Ghirimoldi and Sanchez-Soto (37) found that second-generation female immigrants born in the United States with higher socioeconomic status and educational attainment exhibited significantly greater participation in breast cancer prevention awareness and screenings, reflecting the clear characteristics of SA. Conversely, first-generation female immigrants who demonstrated integrative assimilation tendencies toward American culture, despite possessing adequate educational backgrounds and economic resources, tended to exhibit lower participation in breast cancer prevention awareness and screening. This can be attributed to deep-rooted cultural values and a limited understanding of breast cancer risk. The “healthy immigrant effect” suggests that as immigrants assimilate into their host society, they often experience declining health, which is closely linked to reduced HEU and lower proactive health awareness among FIA immigrants (43). As FIA immigrants age, inadequate HEU can increase the burden of chronic diseases, increasing pressure on social security systems. To address this, this study recommends that health management authorities implement targeted health education services, leverage technology to provide flexible, multilingual online options, and increase awareness through engaging methods such as interactive simulations and bilingual content (44–46).

Third, validating Hypothesis 2c showed that HEU among socioeconomically disadvantaged urban SUA immigrants exceeds that of SSA immigrants, making it the highest among the four assimilation types. This may stem from the fact that health education initiatives in urban Chinese communities primarily target vulnerable populations, focusing on essential topics such as occupational disease prevention, sexually transmitted infections (STIs), reproductive health, tuberculosis prevention, smoking cessation, mental health, chronic disease management, and maternal and child health. These topics are particularly relevant to SUA groups, which typically lack foundational health literacy (47). SUA individuals often live in substandard housing and work in hazardous conditions, increasing their susceptibility to occupational and infectious diseases. To address these risks, community health education programs provide targeted support, such as free health supplies to encourage participation. For instance, STI prevention campaigns distribute contraceptives, while occupational health initiatives offer basic screenings for hypertension and diabetes alongside protective equipment (40). International studies highlight that economic constraints make SUA individuals more likely to participate in free health education programs, as such resources are particularly beneficial (45). Omenka et al. (48) found that SUA African immigrants in the United States, facing barriers like high medical costs, complex healthcare systems, economic hardships, and racial discrimination, actively seek community-based free health education to prevent costly healthcare visits.

However, despite their high HEU, SUA individuals remain constrained by economic and living conditions, which limit their ability to maintain basic health standards. This finding aligns with Pirzada’s et al. (49) study on second-generation Mexican American immigrants, which revealed that despite their knowledge of healthy eating, they often opt for inexpensive, unhealthy food because of financial limitations. Thus, this research suggests that government agencies in host communities should prioritize improving fundamental livelihood issues affecting marginalized migrant populations, including housing conditions, children’s education, and medical insurance, to enable SUA individuals to focus on health management (50).

Furthermore, examining additional variables indicated that several key demographic factors were significantly associated with HEU. These factors include ethnicity, marital status, employment status, educational attainment, and hukou type. Specifically, individuals who are married, self-employed, have higher educational attainment, and have a rural-to-urban hukou type tend to exhibit higher HEU. In contrast, those covered by the UEBMI demonstrated lower HEU, possibly reflecting time constraints among full-time employees, which limits their participation in community health education initiatives. Moreover, individuals who required more than an hour to access healthcare services reported higher HEU. This finding implies that prolonged wait times may motivate individuals to proactively seek health education and independently manage health concerns. Similarly, when migrants encounter difficulties in finances, housing, caregiving for parents in their hometowns, or their children’s education, their participation in HEU tends to decline significantly. This suggests that when facing such challenges, migrants are too preoccupied or lack the time to engage in health education. Regarding social interactions, lower levels of social participation and social integration among migrants were also correlated with reduced HEU. These individuals find it more challenging to adjust to the new environment, limiting their engagement in health education activities, which, despite being a valuable avenue for social engagement, are not fully leveraged.

Moreover, we identified several factors that, while not significantly associated with HEU, warrant further consideration. For example, POR indicates that, in the absence of external interventions, the perceptions and attitudes of four categories of migrants toward health education services remain stable with longer durations of migration, suggesting a more pronounced intergenerational effect. Additionally, among migrants, those with higher education levels, a group that constitutes a very small proportion (e.g., graduate-level, accounting for 0.32%), are estimated to possess greater confidence in their ability to acquire health-related knowledge and maintain health literacy, leading them to perceive a diminished need for health education services.

However, there are few limitations regarding the professionalism and comprehensiveness of the national data used in this study. First, the data were derived from CMDS, which lacks variables related to family structure. Family structure is crucial in shaping immigrants’ socioeconomic status, cultural adaptability, and children’s education, thereby influencing SA outcomes. For instance, families with multiple children may face greater burdens than those with one or no children, potentially affecting parents’ engagement in health education. Second, this study employed a cross-sectional survey design, which limited its ability to comprehensively analyze the dynamic relationship between HEU and its effects. Longitudinal studies to track changes in HEU across generations are valuable and could be expanded to include experimental designs assessing intervention effectiveness, as longitudinal data can continuously observe the differences in assimilation effects across generations. This limitation may introduce biases in guiding health education strategy formulation by government policymakers. Third, this study lacked case studies or anecdotes on assimilation pathways and their impact on health behaviors, as well as thematic analysis of open-ended responses to enrich the qualitative data alongside the quantitative research. Lastly, the data collection methods for HEU and related variables relied on retrospective self-reports, which may introduce recall bias and affect the accuracy of the reported HEU information. Addressing these limitations in future studies will enhance the validity and reliability of findings in this important area of research.

## 5 Conclusion

This study advances research on health management for migrant populations by providing theoretical foundations and practical recommendations for government agencies to design public health strategies tailored to the diverse needs of immigrant groups. It contributes significantly to the literature on SA in healthcare by offering critical insights for global health authorities in shaping immigrant health policies and population management strategies.

Theoretically, this research extends SA theory, traditionally used to examine cultural adaptation pathways among immigrants, to the domain of immigrant health (25). It analyzed HEU patterns among internal migrants in China using official CMDS data. This study enriches SA theory by demonstrating its relevance to intergenerational adaptation, public health management, and health administration within the context of domestic and international population mobility in mainstream societies.

This study investigated HEU among immigrants in China, demonstrating how assimilation processes are shaped by a complex interplay of socioeconomic status, ethnic and racial backgrounds, community environments, and educational attainment. Consequently, immigrants follow various developmental pathways during their assimilation journeys. By applying negative binomial regression analysis, this study revealed that second-generation immigrants exhibit higher HEU than their first-generation counterparts, with the highest HEU levels found among SUA migrants. This finding reflects the Chinese government’s sustained efforts to provide health education to vulnerable migrant populations, encouraging public health management initiatives focused on preventing and managing chronic and infectious diseases in more developed regions. Furthermore, this study contributes to the literature by elucidating the relationship between SA patterns and HEU. This relationship is particularly pertinent for understanding public health management, health administration, disease prevention, and healthcare costs associated with migration from underdeveloped to developed areas. Finally, the analysis of social background variables and their association with HEU underscores the significant role of SA theory in contextualizing the immigrant experience. This study argues that assimilation processes and outcomes should be evaluated in relation to the specific local social contexts into which immigrants are integrated. This perspective highlights the need to consider the nuanced dynamics of social integration when developing policies and programs aimed at enhancing the health and well-being of immigrant populations.

This study offers practical insights for developing countries experiencing migration from underdeveloped to developed regions, emphasizing the importance of cross-cultural integration and adaptation among migrants. Regional disparities in healthcare, education, living conditions, and socioeconomic status complicate the adaptation process, particularly for migrants from less-developed areas. In healthcare, targeted strategies are needed to enhance the positive effects of assimilation while mitigating health declines across various assimilation groups. For the FIA group, health education programs should be flexible and diverse, addressing key barriers such as time constraints, language challenges, and financial constraints. Modern information technology can be leveraged to provide innovative, real-time opportunities for online participation in health education activities. Furthermore, employing diverse educational methods, such as multilingual case studies, simulation training, and stratified teaching, can effectively enhance FIA individuals’ proactive health awareness and engagement in health education (41). For example, Bond et al. (42) developed a web-based e-learning tool incorporating short videos and interactive Q&A scenarios, offering a more engaging and humorous approach to health education. Similarly, Wang et al. (43) created bilingual health education videos in Cantonese and Mandarin, complemented by English subtitles, to support communication between first-generation Chinese-American immigrant women and their second-generation children. Continuous promotion and systematic evaluation of these initiatives are critical to ensuring their long-term effectiveness and impact. For the SUA group, efforts should focus on improving social participation, basic living conditions, and access to employment and education. Providing vocational training can help bridge this social gap by fostering stable employment. Furthermore, enhancing access to healthcare services and health education is crucial for promoting proactive health behaviors. Governments should develop comprehensive health management strategies for migrant populations, including diverse health education programs, to improve health literacy. Encouraging participation in the URRBMI program is vital for long-term health security. Community-based interventions—such as regular disease screenings, low-cost checkups, and establishing health records—can improve access to healthcare, leading to better health outcomes for migrant communities.

## Data Availability

Publicly available datasets were analyzed in this study. This data can be found: https://www.ncmi.cn/phda/dataDetails.do?id=CSTR:A0006.11.A000T.201906.000225.

## References

[1] Zou P Parry M. Strategies for health education in north american immigrant populations. Int Nurs Rev. (2012) 59:482–8. doi: 10.1111/j.1466-7657.2012.01021.x, PMID: 23134131

[2] Lebano A Hamed S Bradby H Gil-Salmerón A Durá-Ferrandis E Garcés-Ferrer J. Migrants’ and refugees’ health status and healthcare in europe: a scoping literature review. BMC Public Health. (2020) 20:1039. doi: 10.1186/s12889-020-08749-8, PMID: 32605605 PMC7329528

[3] Misra S Kwon SC Abraído-Lanza AF Chebli P Trinh-Shevrin C Yi SS. Structural racism and immigrant health in the United States. Health Educ Behav. (2021) 48:332–41. doi: 10.1177/10901981211010676, PMID: 34080482 PMC8935952

[4] Berens E Ganahl K Vogt D Schaeffer D. Health literacy in the domain of healthcare among older migrants in Germany (North Rhine-Westphalia). Findings from a cross-sectional survey. Int J Migr Health Soc Care. (2021) 17:62–74. doi: 10.1108/IJMHSC-09-2019-0078

[5] Finch BK Lim N Rez WPE Do DP. Toward a population health model of segmented assimilation: the case of low birth weight in Los Angeles. Sociol Perspect. (2007) 50:445–68. doi: 10.1525/sop.2007.50.3.445

[6] Ponce-Gonzalez IM Perez K Cheadle AD Jade M Iverson B Parchman ML. A multicomponent health education campaign led by community health workers to increase influenza vaccination among migrants and refugees. J Prim Care Community Health. (2021) 12:627226101. doi: 10.1177/21501327211055627, PMID: 34814785 PMC8640325

[7] Ponce-Gonzalez IM Cheadle AD Parchman ML. Correlation of oral health education by community health workers with changes in oral health practices in migrant populations in Washington state. J Prim Care Community Health. (2021) 12:627279311. doi: 10.1177/21501327211002417, PMID: 33719689 PMC7968011

[8] Khullar D Chokshi DA. Challenges for immigrant health in the Usa-the road to crisis. Lancet. (2019) 393:2168–74. doi: 10.1016/S0140-6736(19)30035-2, PMID: 30981536

[9] Read JG. Does an immigrant health advantage exist among us whites? Evidence from a nationally-representative examination of mental and physical well-being. J Immigr Minor Health. (2024) 26:878–86. doi: 10.1007/s10903-024-01607-4, PMID: 38825664 PMC11412786

[10] Yasenov VI Lawrence D Mendoza FS Hainmueller J. Public health insurance expansion for immigrant children and interstate migration of low-income immigrants. JAMA Pediatr. (2020) 174:22–8. doi: 10.1001/jamapediatrics.2019.4241, PMID: 31738388 PMC6865314

[11] Johns DJ Langley TE Lewis S. Use of social media for the delivery of health promotion on smoking, nutrition, and physical activity: a systematic review. Lancet. (2017) 390:S49. doi: 10.1016/S0140-6736(17)32984-7

[12] Tian Y Luo T Chen Y. The promotional effect of health education on the medical service utilization of migrants: evidence from China. Front Public Health. (2022) 9:818930. doi: 10.3389/fpubh.2021.818930, PMID: 35155362 PMC8831805

[13] Sönmez S Apostolopoulos Y Lemke MK Hsieh YJ. Understanding the effects of covid-19 on the health and safety of immigrant hospitality workers in the United States. Tour Manag Perspect. (2020) 35:100717. doi: 10.1016/j.tmp.2020.100717, PMID: 32834958 PMC7358760

[14] Portes A Fernandez-Kelly P Haller W. Segmented assimilation on the ground: the new second generation in early adulthood. Ethn Racial Stud. (2005) 28:1000–40. doi: 10.1080/01419870500224117

[15] Castro FG Marsiglia FF Kulis S Kellison JG. Lifetime segmented assimilation trajectories and health outcomes in latino and other community residents. Am J Public Health. (2010) 100:669–76. doi: 10.2105/ajph.2009.167999, PMID: 20167890 PMC2836354

[16] Ramírez AS Wilson MD Soederberg ML. Segmented assimilation as a mechanism to explain the dietary acculturation paradox. Appetite. (2022) 169:105820. doi: 10.1016/j.appet.2021.105820, PMID: 34843752 PMC8944242

[17] Lubbers MJ Molina JEL Mccarty C. How do migrants’ processes of social embedding unfold over time?. Global networks (2021) 21:529–50. doi: 10.1111/glob.12297

[18] Feliciano C Lanuza Y. An immigrant paradox? Contextual attainment and intergenerational educational mobility. Am Sociol Rev. (2017) 82:211–41. doi: 10.1177/0003122416684777

[19] Umaña-Taylor AJ Kornienko O McDermott ER Motti-Stefanidi F. National identity development and friendship network dynamics among immigrant and non-immigrant youth. J Youth Adolesc. (2020) 49:706–23. doi: 10.1007/s10964-019-01181-1, PMID: 31865472

[20] Yajie Z Xia W. Does accepting public health education improve the health status of floating population: an empirical analysis based on the data of the China migrants dynamic survey in 2018. J Hunan Agric Univ. (2020) 21:61–7. doi: 10.13331/j.cnki.jhau(ss).2020.05.008

[21] Hu X Cook S Salazar MA. Internal migration and health in China. Lancet. (2008) 372:1717–9. doi: 10.1016/S0140-6736(08)61360-4, PMID: 18930533 PMC7135200

[22] Hagos RM Hamilton TG. Beyond acculturation: health and immigrants' social integration in the United States. J Health Soc Behav. (2024) 65:356–80. doi: 10.1177/00221465241231829, PMID: 38504618

[23] Lee SK Sobal J Frongillo EJ. Acculturation and health in korean americans. Soc Sci Med. (2000) 51:159–73. doi: 10.1016/s0277-9536(99)00446-3, PMID: 10832565

[24] Flannery W Reise S Yu JJ. An empirical comparison of acculturation models. Personal Soc Psychol Bull. (2001) 27:1035–45. doi: 10.1177/0146167201278010

[25] Karimi A Wilkes R. Classic, segmented-, or neo-assimilation, which theory to use? A scientific-method investigation. Int Migr Rev. (2023). doi: 10.1177/01979183231205560

[26] Jang Y Park J Choi EY Cho YJ Park NS Chiriboga DA. Social isolation in asian americans: risks associated with socio-demographic, health, and immigration factors. Ethn Health. (2022) 27:1428–41. doi: 10.1080/13557858.2021.1881765, PMID: 33550840

[27] Saadi A Sanchez MU Franco-Vasquez A Inkelas M Ryan GW. Assessment of perspectives on health care system efforts to mitigate perceived risks among immigrants in the United States: a qualitative study. JAMA Netw Open. (2020) 3:e203028. doi: 10.1001/jamanetworkopen.2020.3028, PMID: 32301990 PMC7165299

[28] Porru S Baldo M. Occupational health and safety and migrant workers: has something changed in the last few years? Int J Environ Res Public Health. (2022) 19:9535. doi: 10.3390/ijerph19159535, PMID: 35954890 PMC9367908

[29] Farley R Alba R. The new second generation in the United States. Int Migr Rev. (2002) 36:669–701. doi: 10.1111/j.1747-7379.2002.tb00100.x

[30] Portes A Rumbuat R. The forging of a new america: lessons for theory and policy. In: Ethnicities: Children of Immigrants in America (2001). doi: 10.1525/california/9780520230118.003.0010

[31] Akay A Bargain O Zimmermann KF. Relative concerns of rural-to-urban migrants in China. J Econ Behav Organ. (2012) 81:421–41. doi: 10.1016/j.jebo.2011.12.006

[32] Stellefson M Paige SR Chaney BH Chaney JD. Evolving role of social media in health promotion: updated responsibilities for health education specialists. Int J Environ Res Public Health. (2020) 17:1153. doi: 10.3390/ijerph17041153, PMID: 32059561 PMC7068576

[33] Ikotun AM Ezugwu AE Abualigah L Abuhaija B Heming J. K-means clustering algorithms: a comprehensive review, variants analysis, and advances in the era of big data. Inf Sci. (2023) 622:178–210. doi: 10.1016/j.ins.2022.11.139

[34] Norredam M Nielsen SS Krasnik A. Migrants' utilization of somatic healthcare services in europe--a systematic review. Eur J Pub Health. (2010) 20:555–63. doi: 10.1093/eurpub/ckp195, PMID: 20040522

[35] Tarnutzer S Bopp M. Healthy migrants but unhealthy offspring? A retrospective cohort study among italians in Switzerland. BMC Public Health. (2012) 12:1104. doi: 10.1186/1471-2458-12-1104, PMID: 23259829 PMC3552682

[36] Zhang X Wang Z Li T. The current status of occupational health in China. Environ Health Prev Med. (2010) 15:263–70. doi: 10.1007/s12199-010-0145-2, PMID: 21432554 PMC2921040

[37] Ghirimoldi F Sanchez-Soto G. Immigrant assimilation and profiles of breast cancer screening behaviors among u. s. Immigrant women. Health Care Women Int. (2021) 42:213–34. doi: 10.1080/07399332.2020.1797034, PMID: 32779966

[38] Castañeda H Holmes SM Madrigal DS Young ME Beyeler N Quesada J. Immigration as a social determinant of health. Annu Rev Public Health. (2015) 36:375–92. doi: 10.1146/annurev-publhealth-032013-182419, PMID: 25494053

[39] Zhang F Shi X Zhou Y. The impact of health insurance on healthcare utilization by migrant workers in China. Int J Environ Res Public Health. (2020) 17:1852. doi: 10.3390/ijerph17061852, PMID: 32178431 PMC7143864

[40] Ichou M Wallace M. The healthy immigrant effect: the role of educational selectivity in the good health of migrants. Demogr Res. (2019) 40:61–94. doi: 10.4054/DemRes.2019.40.4

[41] Stark AL Geukes C Dockweiler C. Digital health promotion and prevention in settings: scoping review. J Med Internet Res. (2022) 24:e21063. doi: 10.2196/21063, PMID: 35089140 PMC8838600

[42] Bond SE Crowther SP Adhikari S Chubaty AJ Yu P Borchard JP. Design and implementation of a novel web-based e-learning tool for education of health professionals on the antibiotic vancomycin. J Med Internet Res. (2017) 19:e93. doi: 10.2196/jmir.6971, PMID: 28360025 PMC5391435

[43] Wang JH Liang W Schwartz MD Lee MM Kreling B Mandelblatt JS. Development and evaluation of a culturally tailored educational video: changing breast cancer-related behaviors in Chinese women. Health Educ Behav. (2008) 35:806–20. doi: 10.1177/1090198106296768 17602099

[44] Xue L Fan H Guo J. Current situation of health education and its influencing factors among migrant population. Chin J Health Educ. (2017) 33:771–4. doi: 10.16168/j.cnki.issn.1002-9982.2017.09.001

[45] Huang Y Miao L Lyu B. Urban public health education services, health status, and increased fertility intentions of the rural migrant population. Reprod Health. (2023) 20:108. doi: 10.1186/s12978-023-01648-2, PMID: 37488609 PMC10367397

[46] Zhao Q Song M Wang H. Voting with your feet: the impact of urban public health service accessibility on the permanent migration intentions of rural migrants in China. Int J Environ Res Public Health. (2022) 19. doi: 10.3390/ijerph192214624 PMC9691094 36429343

[47] Zhang L Liu S Zhang G Wu S. Internal migration and the health of the returned population: a nationally representative study of China. BMC Public Health. (2015) 15:719. doi: 10.1186/s12889-015-2074-x, PMID: 26215980 PMC4517553

[48] Omenka OI Watson DP Hendrie HC. Understanding the healthcare experiences and needs of african immigrants in the United States: a scoping review. BMC Public Health. (2020) 20:27. doi: 10.1186/s12889-019-8127-9, PMID: 31914960 PMC6950921

[49] Pirzada A Cai J Cordero C Gallo LC Isasi CR Kunz J. Risk factors for cardiovascular disease: knowledge gained from the hispanic community health study/study of latinos. Curr Atheroscler Rep. (2023) 25:785–93. doi: 10.1007/s11883-023-01152-9, PMID: 37773246 PMC12344663

[50] Fei C Zhu Y Jiang L Zhou H Yu H. Social integration, physical and mental health and subjective well-being in the floating population-a moderated mediation analysis. Front Public Health. (2023) 11:1167537. doi: 10.3389/fpubh.2023.1167537, PMID: 37483925 PMC10356978

