# Supplementary material for: Exploring segmented assimilation theory in health education utilization and its influencing factors among internal migrants in China: insights from the 2017 China migrants dynamic survey
Source: Front Public Health. 2025 Jan 8;12:1529736. doi: 10.3389/fpubh.2024.1529736 (PMC11751069; doi:10.3389/fpubh.2024.1529736)
Supplement: Supplementary file 1 [file Supplementary_file_1.doc]

**Appendix 1: Poisson Regression Analysis of Health Education Utilization Among Internal Migrants in Mainland China**

| **Variables** | **HEU- IRR value** | **95% Confidence Interval** | **P-Value** |
| --- | --- | --- | --- |
| **Segmentation assimilation type** |  |  |  |
| SSA type | 1.000 |  |  |
| FCA type | 0.928 | （0.839-1.028） | 0.152 |
| FIA type | 0.931** | （0.887-0.977） | 0.004 |
| SUA type | 1.096** | （1.035-1.161） | 0.002 |
| **POR** |  |  |  |
| 1-5 years | 1.000 |  |  |
| 5-10 years | 1.042 | (0.991,1.095) | 0.107 |
| 10-20 years | 0.978 | (0.915-1.046) | 0.520 |
| More than 20 years | 1.099 | (0.959-1.260) | 0.175 |
| **Age** | 0.999 | （0.996-1.003） | 0.883 |
| **Han nationality** |  |  |  |
| No | 1.000 |  |  |
| Yes | 0.887** | (0.814-0.966) | 0.002 |
| **Marital status** |  |  |  |
| Married | 1.000 |  |  |
| Unmarried | 0.872*** | (0.813-0.936) | <0.001 |
| **Employment status** |  |  |  |
| An employee with a fixed employer | 1.000 |  |  |
| An employee without a fixed employer | 1.056 | (0.952-1.172) | 0.304 |
| Employer | 0.998 | (0.906-1.100) | 0.971 |
| Self-employed worker | 1.098*** | (1.041-1.159) | 0.001 |
| Others | 1.079 | (0.899-1.295) | 0.414 |
| **Educational attainment** |  |  |  |
| Illiteracy | 1.000 |  |  |
| Primary school | 1.373 | (1.047-1.833) | 0.017 |
| Junior high school | 1.508** | (1.18-2.048) | 0.002 |
| High school/technical secondary school | 1.608*** | (1.255-2.197) | <0.001 |
| Junior college | 1.659*** | (1.272-2.254) | <0.001 |
| Undergraduate | 1.629*** | (1.249-2.252) | <0.001 |
| Graduate | 1.113 | (0.640-1.754) | 0.631 |
| **Hukou type** |  |  |  |
| Rural | 1.000 |  |  |
| Non-agricultural | 0.999 | (0.930-1.073) | 0.982 |
| Agricultural to residential | 1.139** | (1.044-1.243) | 0.004 |
| Non- agricultural to residential | 0.920 | (0.743-1.139) | 0.442 |
| Residential | 0.941 | (0.826-1.072) | 0.359 |
| **Health insurance type** |  |  |  |
| **URRBMI** |  |  |  |
| No | 1.000 |  |  |
| Yes | 1.098 | (0.959-1.187) | 0.055 |
| **URBMI** |  |  |  |
| No | 1.000 |  |  |
| Yes | 1.141 | (1.010-1.265) | 0.012 |
| **UEBMI** |  |  |  |
| No | 1.0000 |  |  |
| Yes | 0.907** | (0.815-0.954) | 0.008 |
| **FI** |  |  |  |
| No | 1.000 |  |  |
| Yes | 1.083 | (0.975-1.432) | 0.333 |
| **Time of Access to health care** |  |  |  |
| Within 15 minutes | 1.000 |  |  |
| 15-30 minutes | 1.032 | (0.971-1.097) | 0.301 |
| 30-60 minutes | 1.021 | (0.887-1.174) | 0.776 |
| More than an hour | 1.503*** | (1.251-1.806) | <0.001 |
| **Social integration** | 1.123*** | (1.104-1.144) | <0.001 |
| **Social participation** | 1.071*** | (1.059-1.085) | <0.001 |
| **Whether to establish a health record** |  |  |  |
| No | 1.000 |  |  |
| Yes | 1.494*** | (1.040 - 1.374) | <0.001 |
| **Whether to encounter problems in local place** |  |  |  |
| No | 1.000 |  |  |
| Yes | 0.995*** | (1.435 - 1.556) | 0.000 |
| Constant | 1.299 | (0.945- 1.787) |  |
| Observed value | 6184 |  |  |
| Pseudo R-squared | 0.0804 |  |  |
| Fitness of model | Pilsenka square value | 1760.68 |  |
|  | P value | 0.000 |  |

**p < 0.05: * p < 0.01: ** p < 0.001: *****

**IRR =Incidence Rate Ratio**
